# Treating heterogeneity and uncertainty in data integration: study on Brazilian healthcare databases.

**DOI:** 10.23889/ijpds.v1i1.313

**Published:** 2017-04-19

**Authors:** Marcos Barreto, Spiros Denaxas

**Affiliations:** Federal University of Bahia (UFBA); Farr Institute of Health Informatics Research London

## Background and aims

Data integration comprises methods and tools to aggregate data from disparate sources to various purposes. Heterogeneity and uncertainty are technical challenges in this field. The first involves different data representation or meaning, while the second refers to incomplete data or the expectancy that a data item exists in a data source. Our goal is to design and validate a data integration model and computing tools able to address both problems. Such model and tools will support the setup of a population-based cohort comprised by 100 million individuals and the generation of data marts (domain-specific data) to be used in epidemiological studies within an ongoing cooperation Brazil-UK. Such studies will assess the impact of a conditional cash transfer programme (PBF – Bolsa Família) on the occurrence, severity and mortality of several diseases and health problems (hospitalization, mortality, child health etc) over this cohort.

## Approach

We propose a three-dimensional data model to aggregate information on the cohort, exposition (payments received during the observed period) and health outcomes. We treat heterogeneity based on our existing probabilistic linkage pipeline that provides data quality assessment, data conditioning (standardization, cleansing, blocking, and anonymization), two methods for probabilistic record matching, and accuracy assessment. Through this pipeline, we are able to probabilistically link records from PBF, Cadastro\'Unico (CADU - socioeconomic information) and healthcare databases from the Unified Health System (SUS). Uncertainty is modeled through ``possible worlds'', which represent a data instance (record) with a corresponding probability. There exist 2\^records possible worlds with the probability distribution being the product of record probabilities. We map the most probable relationships between all the databases involved and create some simulation scenarios in order to validate them. We are seeking for a good balance between the set of possible worlds, not overextending the possibilities, and their proximity to a real scenario. 

## Results

The current implementation comprises the linkage of the 2011 extraction of CADU and healthcare databases to populate the proposed model. Such linkage provides timely execution (up to 9 hours depending on the databases) with high accurate data marts (over 95% of true positive matched pairs) for samples with increasing size (from 1,447,512 to 12,036,010 records).

## Conclusions

Our model is able to treat heterogeneity aspects present in huge databases. Our tools provide timely execution of probabilistic linkage with high accuracy. We started to model uncertainty in order to perform simulations and decide how to incorporate it in our model.

